# Cancer Stem Cells and the Tumor Microenvironment in Gastric Cancer

**DOI:** 10.3389/fonc.2021.803974

**Published:** 2022-01-03

**Authors:** Ying Yang, Wen-Jian Meng, Zi-Qiang Wang

**Affiliations:** Department of Gastrointestinal Surgery, West China Hospital, Sichuan University, Chengdu, China

**Keywords:** gastric cancer, cancer stem cells (CSC), tumor microenvironment, mesenchymal stem cells, cancer associated fibroblasts (CAFs), tumor-associated macrophages (TAMs)

## Abstract

Gastric cancer (GC) remains one of the leading causes of cancer-related death worldwide. Cancer stem cells (CSCs) might be responsible for tumor initiation, relapse, metastasis and treatment resistance of GC. The tumor microenvironment (TME) comprises tumor cells, immune cells, stromal cells and other extracellular components, which plays a pivotal role in tumor progression and therapy resistance. The properties of CSCs are regulated by cells and extracellular matrix components of the TME in some unique manners. This review will summarize current literature regarding the effects of CSCs and TME on the progression and therapy resistance of GC, while emphasizing the potential for developing successful anti-tumor therapy based on targeting the TME and CSCs.

## Introduction

Gastric cancer (GC) is the fifth most commonly diagnosed cancer and the fourth leading cause of cancer-associated mortality with an estimated more than one million new cases and 769,000 deaths worldwide in 2020 (1). Despite advancements in clinical treatment and technologies, the prognosis of GC remains poor, mainly due to relapse, metastasis and therapy resistance, and the median survival of patients with advanced GC is less than one year (2). Cancer stem cells (CSCs) are a minor subpopulation of uniquely tumorigenic cells exhibiting the capacity for self-renewal, unlimited proliferating and maintenance of a relatively dormant state. Since the first identification of CSCs in acute myeloid leukemia (3), increasing evidence suggests that CSCs may be responsible for tumor (including GC) progression, relapse, metastasis and therapy resistance (4–6).

**Figure 1:**
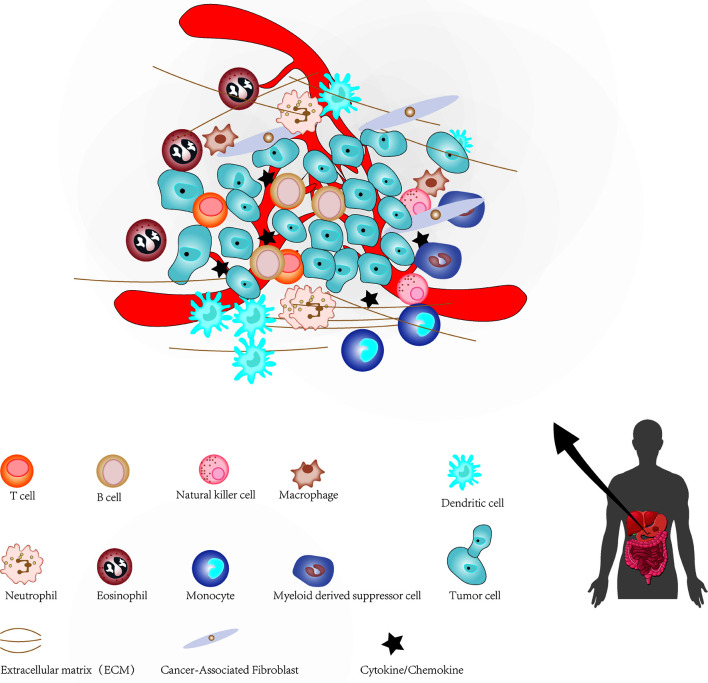
The major constituents of the tumor microenvironment in gastric cancer.

The cellular environment in which tumor cells reside is called the tumor microenvironment (TME), which consists of cellular and non-cellular components. It includes many types of stromal cells (fibroblasts, lymphocytes, macrophages, and endothelial cells), immune cells (such as T and B lymphocytes), and extracellular components (for instance: cytokines, growth factors, hormones and extracellular matrix), which surround tumor cells and are nourished by blood vessels around the tumor (**Figure 1**). The TME provides a suitable living environment for cancer cells to develop, escape from host immune surveillance and resist to anticancer drugs (7–10). With the continuous progress in the study of CSCs and TME, the microenvironment of CSC has gradually entered the vision of researchers. CSCs microenvironment (CSCs niche) is a special microenvironment for the survival of CSCs, which can regulate the characteristics of CSCs *via* cell-to-cell contact and secreted factors (11). Therefore, a precise and meticulous understanding of CSCs, TME and the relationship between them in GC will have a profound impact on the treatment of GC in the future. This review summarized current findings regarding the role of CSCs and TME in the progression of GC, which may facilitate the understanding of CSCs and TME of GC, as well as provide a potential therapeutic strategy based on targeting TME and CSCs for GC.

## Identification and Isolation of CSCs

The study of CSCs may play a critical role in eradicating tumors and solving clinical problems such as tumor recurrence and distant metastasis. However, the number of CSCs in cancer cells is extremely small (typically <1% in solid tumors) (12). Therefore, the main difficulty in studying CSCs is how to identify and isolate them from a large number of tumor cells. At present, the most efficacious and commonly used method to isolate CSCs from a large number of tumor cells is by using specific cell surface markers of CSCs.

CSCs can be isolated and identified by combining specific cell surface markers of CSCs with corresponding monoclonal antibodies or fluorescein markers, and then applying some isolation techniques such as flow cytometry and magnetic-activated cell sorting. Therefore, finding specific and effective cell surface markers of CSCs is critical for isolating and identifying CSCs. An increasing number of evidence has confirmed the existence of some specific cell surface markers in gastric CSCs (GCSCs). CD44 was the first confirmed potential GCSCs-specific cell surface marker. Takaishi et al. (13) found a considerable number of CD44 (+) cells in GC cell lines (MKN-45, MKN-74, and NCI-N87), and these CD44 (+) cells showed spheroid colony formation in serum-free media *in vitro*. And when these cells were injected into the stomach and skin (around 30,000 cells per site) of severe combined immunodeficiency (SCID) mice, the significant tumorigenic ability *in vivo* was showed. However, only about 5% of the CD44 (+) cells were ultimately identified as true CSCs. In addition, CD44 is also widely expressed by nonmalignant tumors. Hence, it seems unlikely that a single cell surface marker of CD44 could detect all cells with the characteristics of GCSCs. Lau et al. (14) identified CD44v8-10 (the predominant CD44variant expressed in GC cells) as another potential cell surface marker of GCSCs. The results showed that the expression of CD44v8-10 was significantly upregulated in gastric tumor sites and that exogenous expression of CD44v8-10 contributed to tumor initiation in immunocompromised mice, possibly by improving oxidative stress defense. As a family of intracellular enzymes, aldehyde dehydrogenase (ALDH) enzymes are responsible for cell differentiation, detoxification and drug resistance through the oxidation of cellular aldehydes. Katsuno et al. (15) demonstrated that ALDH1(+) cells possess the characteristics of CSCs, accounting for about 5-8% of the human diffuse-type gastric carcinoma cells and displaying a higher tumorigenicity than ALDH1(-) cells. These findings indicate that ALDH1 is a potential specific cell surface marker for GCSCs. Numerous other molecules or proteins have also been suggested as potential cell surface markers for GCSCs. For instance, Jiang et al. (16). found that CD90(+) cells possessed an increased capacity of tumorigenicity *in vivo* compared with CD90(-) cells, and the expression level of CD90(+) cells was positively correlated with the tumorigenicity of GC cells *in vivo*. In addition, a combination therapy of trastuzumab with conventional chemotherapy is able to suppress tumor growth by reducing the proportion of CD90 (+) cells. These findings suggest that CD90 may be a potential cell surface marker for GCSCs. Ohkuma et al. (17) have investigated the role of CSCs in gastric adenocarcinoma using MKN-1 cells, which showed that CD71 (−) cells were more tumorigenic than CD71 (+) cells in the gastric adenosquamous carcinoma model and that most CD71 (−) cells were dormant (G1/G0 cell cycle phase) and resistant to 5-FU. These characteristics of CD71 (−) cells were highly consistent with the characteristics of CSCs. Wenqi et al. (18) demonstrated that the epithelial cellular adhesion molecule (EpCAM) was overexpressed by gastric cancer cells and down-regulation of EpCAM was able to inhibit tumor formation, reduce cell proliferation, and reduce the proportion of cells in a relatively dormant state. However, there are still some limitations and controversies about the use of these molecules as separate markers. Combining several molecular as cell surface markers to improve the ability of specifically identifying and isolating GCSCs has also been confirmed by some studies. For instance, CD44 was combined with other molecules as a specific cell surface marker for GCSCs, including CD24 (19), CD54 (20) and EpCAM (21). **Table 1** summarizes the current specific cell surface markers for GCSCs.

**Table 1:** Cell surface markers of gastric cancer stem cells (GCSCs).

GCSCs surface marker	Characteristic of stem cell	Reference
CD44(+)	Tumorigenicity, self-renewal, multipotent differentiation, chemoresistance, colony-forming ability	(13)
CD44v8-10	Tumorigenicity,	(14)
ALDH	Chemoresistance, self-renewal, colony-forming ability, generate heterogeneity	(15, 22)
CD90	Tumorigenicity, self-renewal	(16)
CD71 (−)	Tumorigenicity, self-renewal, relatively dormant state	(17)
EpCAM	Tumorigenicity, relatively dormant state	(18)
CD44(+)/CD24(+)	Tumorigenicity, self-renewal, multipotent differentiation	(19)
CD44(+)/CD54(+)	Tumorigenicity, self-renewal	(20)
EpCAM(+)/CD44(+)	Tumorigenicity, chemoresistance	(21)

In addition to the isolation of CSCs by cell surface markers, some characteristics of CSCs also have been applied to isolate and identify CSCs. In serum-free medium supplemented with growth factors, most non-CSCs cannot survive, while CSCs can survive and maintain their self-renewal characteristics. Therefore, serum-free medium containing growth factors can enrich and isolate CSCs, and Li et al. (23) used serum-free medium to enrich and isolate potential GCSCs. Side population (SP) cell isolation also can be applied to sort and enrich GCSCs by taking advantage of efflux characteristics of CSCs for Hoechst33342(a nucleic acid dye) (24, 25). Traditional two-dimensional (2D) cell cultures do not mimic TME *in vivo* due to the lack of cell-extracellular matrix interactions. Animal models may also not adequately mimic the characteristics of human cancers. Therefore, the 3-dimensional (3D) culture system, which can better simulate *in vivo* cancer environment, has been widely used in cancer research. In addition, 3D culture systems can simulate the TME of CSCs by controlling the mechanical properties of materials and then effectively isolate CSCs. Recently, increasing studies have also used 3D culture systems to isolate and culture GCSCs (26–30).

With the continual development of single cell technologies, it is possible to identify CSCs from cancer cells. CSCs are usually difficult to isolate owing to their low abundance and similarity to other stem cells. Single-cell sequencing technologies are able to detect extremely trace amounts of nucleic acid sequences, which may assist in the identification and study of CSCs (31). For instance, Velten et al. (32) successfully identified leukemic stem cells from acute myeloid leukemia by clonal tracking from single-cell transcriptomics. Yang et al. (33) performed single-cell RNA sequencing of 59 cells from three bladder cancer samples, and finally found six key modifier genes (ETS1, GPRC5A, MKL1, PAWR, PITX2, and RGS9BP) in bladder CSCs that have never been reported. However, with the wide application of single-cell sequencing in the study of tumors, a large quantities of single-cell genomics data also makes it tricky to study. Therefore, Song et al. (34, 35) developed two research models of single-cell genomics data (single-cell Latent-variable Mode and Single-Cell Graph Convolutional Network) for better mining and understanding these data, which will aid in the understanding the complex mechanisms of cancer and CSCs. However, the high price of single-cell sequencing and the complexity of sample handling and operational procedures limit its application, and current studies identifying GCSCs by single-cell sequencing have not been reported.

## The TME in GC

The TME favors the survival of tumor cells and provides an excellent shelter for them to escape host immune surveillance and resist anti-tumor drugs. Meanwhile, cancer cells in the TME can also affect and change their surrounding cells in an autocrine and a paracrine manner to maintain the TME required for the survival of cancer cells. This section will summarize the effects of the main components of TME on GC and the features of gastric TME.

### Cancer Associated Fibroblasts

**Figure 2:**
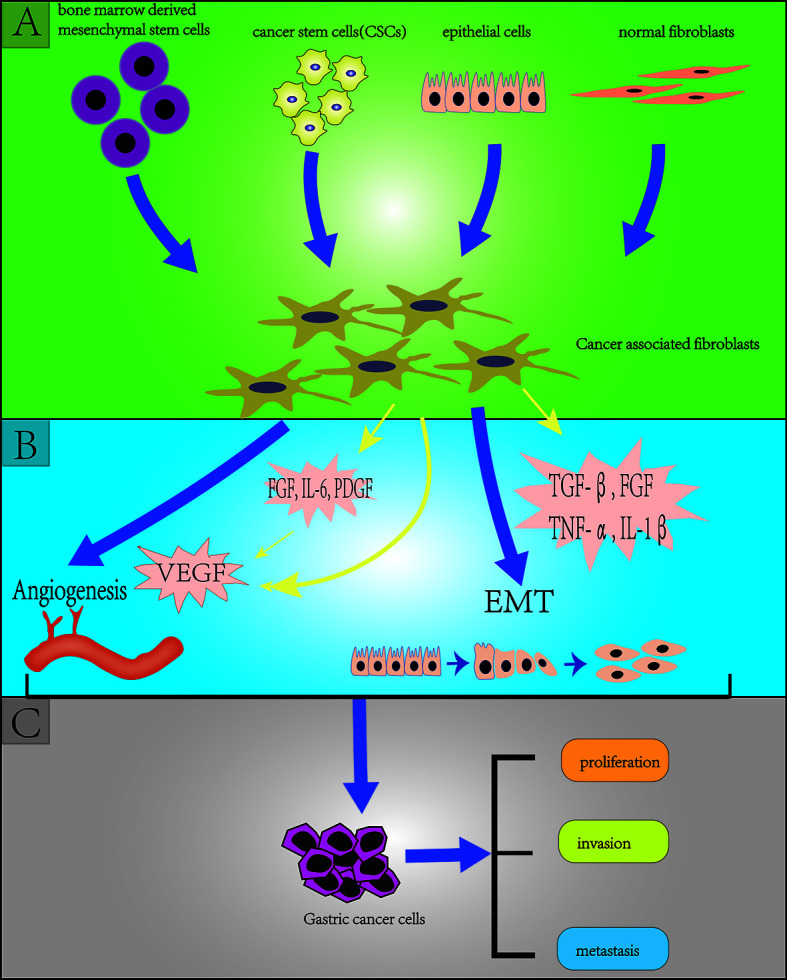
Section **(A)** (green background) indicates the origins of cancer-associated fibroblasts (CAFs) including bone marrow derived mesenchymal stem cells, cancer stem cells, endothelial cells (through endothelial mesenchymal transition: EMT), normal fibroblasts; Section **(B)** (blue background) indicates the effect of CAFs on gastric cancer stem cells (GCSCs) including angiogenesis and EMT; Section **(C)** (grey background) indicates the effect of (GCSCs) on gastric cancer cells including proliferation, invasion and metastasis.

Cancer associated fibroblasts (CAFs) are the most abundant stromal cells in the TME, accounting for about 50% of the total number of tumor tissue cells (36). CAFs can promote tumor development, proliferation, drug resistance, invasion and metastasis through direct contact or secretion of a variety of cytokines and metabolites in a paracrine manner, thereby affecting the prognosis of tumors. There are several controversies about the origins of CAFs, but an increasing number of evidence shows that CAFs originate from a variety of cells, such as bone marrow-derived cells, CSCs, epithelial cells through epithelial-mesenchymal transformation (EMT) and normal fibroblasts. The diversity of origins of CAFs also contributes to the heterogeneity of CAFs (37–40) (**Figure 2**). CAFs not only provide physical support for epithelial cells in TME, but also are key factors for EMT of GC cells (41). EMT is a pathological process closely related to tumor invasion and metastasis. The main changes were that the cells changed from closely arranged epithelial cells to loosely structured interstitial cells, which weakened the adhesion between cells and increased the invasion and metastasis of the tumor. Angiogenesis is considered to be critical for tumor proliferation and metastasis, and vascular endothelial growth factor (VEGF) plays a crucial role in promoting angiogenesis. In GC cells, CAFS has been shown to promote angiogenesis by secreting FGF, IL-6, PDGF and VEGF and promote EMT by secreting transforming growth factor beta (TGF-β), FGF, TNF-α, and IL-1β, in turn leading to proliferation, invasion and metastasis of GC (39, 42–49)(**Figure 2**).

CSCs have been shown to be capable of converting mesenchymal stem cells (MSCs) into CAFs in GC *via* exosome-mediated TGF- transfer and activation of TGF-/Smad pathways (50). In turn, CAFs are also able to maintain the stem-like properties of GC, promote the progression of GC and predict the prognosis of GC patients using the characteristics of CAFs. Hasegawa et al. (42) showed that CAFs may be able to maintain the stemness of sclerosing GC cells through TGF-β signaling. And Spondin-2 secreted by CAFs in GC is positively correlated with peritoneal dissemination, tumor size and poor prognosis of GC (51). Ishimoto et al.  *(* 52) isolated CAFs and adjacent non-cancer fibroblasts from resected specimens of 110 patients with diffuse-type GCs (DGCs) to investigated the characteristics and functions of CAFs in DGCs by analyzing the features of their genome and gene expression patterns. They found that DGCs cells cultured with CAFs were also more aggressive and invasive *in vitro* than those not cultured with CAFs. Further work using quantitative reverse PCR revealed that the expression of the Rhomboid 5 Homolog 2 (RHBDF2) gene associated with TGF-β1 activity was increased in DGC cells, and increased expression of RHBDF2 gene was observed after incubation of non-cancer fibroblasts with interleukin 1 alpha (IL-1α), IL-β or TNF, secreted by DGCs. In view of the above findings, it was concluded that CAFs were able to activate TGF-β1 signaling by increasing the expression of RHBDF2. And the activation of TGF-β1 was demonstrated to increase the motility and invasiveness of GC cells. Fibroblast activating protein (FAP) is a member of the TME, Wen et al. (53) found that overexpression of FAP was negatively correlated with the survival rate of GC patients, and FAP combined with CAFs could promote the proliferation and invasiveness of GC cells and induce the development of chemoresistance of GC cells *in vitro*. In a xenograft model of GC, combined targeted inhibition of FAP and CAFs enhanced the antitumor immunity of immune checkpoint inhibitors. In addition, the characteristics of CAFs can also be used to predict the prognosis of GC patients and estimate the response of GC patients to clinical immunotherapy. Zheng et al. (54) constructed a 4-gene (COL8A1, SPOCK1, AEBP1, and TIMP2) prognostic CAFs model by analyzing mRNA expression and clinical information of GC samples from GEO (Gene Expression Omnibus) and TCGA (Cancer Genome Atlas) databases. The results showed a positive correlation between CAFs risk score and stromal and CAFs infiltrations in GC, and patients in the high-CAF-risk group were less likely to respond to immunotherapy.

### Tumor-Associated Macrophages

The most abundant immune cells in the TME are composed of macrophages or monocytes, which are called tumor-associated macrophages (TAMs). TAMs promote tumor progression by secreting a variety of factors, including growth factors, chemokines, cytokines, proteases and so on. Monocytes or macrophages are usually polarized into two main types: M1 and M2 (55). M1 macrophages enhance the function of T cells by releasing proinflammatory cytokines, such as TNF-α, IL-1 and IL-12 and participate in type I helper T cell (Th) responses, which are crucial components involved in inflammatory responses and anti-tumor immunity (56). TAMs frequently exhibit an M2-like phenotype in the microenvironment of cancer, including GC, and express anti-inflammatory cytokines such as IL-10, TGF-β and arginase (57). These anti-inflammatory cytokines inhibit T cell-mediated anti-tumor immunity and provide tumors with an immunosuppressive microenvironment, allowing tumors to evade host immune surveillance and promote tumor growth and metastasis (58). In addition, Oishi et al. (59) demonstrated that intraperitoneal presentation of M2-polarized macrophages was able to inhibit T cell proliferation *in vitro*. In a mouse model of GC, TAMs were demonstrated to be able to secrete the proinflammatory factor TNF-α, which contributes to the formation and development of GC by activating the Wnt signaling pathway (60, 61). Yamanaka et al. (62) showed that IL-1β secreted by TAMs of GC could increase the invasiveness of GC cells by activating NF-κB and expressing MMP-9. In addition, TAMs can also promote tumor angiogenesis and provides nutrition for tumor growth. Some studies have shown that the level of TAMs is closely associated with the number of blood vessels surrounding the GC cells. Ohta et al. (63) found that expression level of monocyte chemoattractant protein-1 (MCP-1) was significantly increased compared with negative tumors in human GC cell lines, and its expression level was closely related to the secretion of VEGF. And the counts of TAMs are positively correlated with the counts of vessel. They concluded that MCP-1 induced by human GC cells may promote angiogenesis of GC cells by recruiting and activating TAMs. Then, Kuroda et al. (64) further confirmed that MCP-1 also has a similar effect and mechanism (by activating and recruiting TAMs) in a mouse xenograft model of GC. In addition to promoting angiogenesis in GC through MCP-1, TAMs may also directly promote angiogenesis and lymphangiogenesis of GC possibly by enhancing the expression of VEGF and VEGF-C (65).

### Mesenchymal Stem Cells

As an important part of the TME, MSCs play a key role in the process of tumor development, including tumor neovascularization, metastasis, maintenance of the stemness of CSCs and the formation of an immunosuppressive TME by activating signaling pathway and secreting a variety of regulatory factors (66). During the growth of GC, MSCs are recruited into the TME of GC, and they are able to alter the TME and promote tumor growth by secreting a variety of factors. GC-derived MSCs (GC-MSCs) were reported to enhance the proliferation, migration, and promotion of angiogenesis of GC cells by secreting considerable the proinflammatory cytokine interleukin-8 (IL-8) (67). IL-15 secreted by GC-MSCs enhances the stem-like properties of GC cells, induces EMT of GC cells which in turn promotes migration and metastasis of GC cells by upregulating Tregs (Regulatory T cells) ratio and programmed cell death protein-1 expression in CD4 + T Cell (68). Huang et al. (69) found that PDGF-DD secreted by GC-MSCs was capable of promoting the migration and proliferation of GC cells *in vitro* and *in vivo* by phosphorylating PDGF-β. Wang et al. (70) found that GC-MSCs were able to significantly promote the growth and migration of HGC-27 and increase microRNA-221 expression through paracrine secretion. In addition, inhibiting the expression of IL-8, PDGF-DD and microRNA-221 were all able to block its tumor-supporting role on GC cells. Recent studies have demonstrated that MSCs are crucial in the progression of GC. GC-MSCs can promote the growth of GC cells and the polarization of macrophages in the GC microenvironment to the M2 type by considerable secretion of IL-6 and IL-8, and M2 type macrophages can promote GC metastasis by promoting the EMT of GC cells (71). The role of GC-MSCs in promoting GC metastasis and EMT of GC cells was also confirmed in another study (72). In TME, bone marrow-derived MSCs (BM-MSCs) were able to produce CXCL16 through Ror2-mediated signaling. While CXCL16 could induce expression of Ror1 in MKN45 cells, thereby promoting the progression of GC by activating the CXCR6-STAT3 signaling pathway (73). In addition, Wnt5a-Ror2 signaling in BM-MSCs has been shown to promote the proliferation of GC cells (74).

### Tumor-Infiltrating Lymphocytes

Recently, the effect of tumor-infiltrating lymphocytes (TILs) on GC has also been reported. TILs refer to lymphocytes that leave the blood and enter the tumor, which are a major component of the TME, including CD8 + T cells, CD4 + T cells, B lymphocytes and natural killer (NK) cells (75). CD8 + T cells, also known as cytotoxic T cells (CTL), are recognized as the main anti-tumor immune effector cells. The subsets of CD4 + T cells are represented by Th1, Th2 and Treg. Th1 cells, which secrete IL-2 and interferon, play a crucial role in activating and promoting the proliferation of CD8 + T cell and NK cell. Th2 enhances humoral immunity by secreting cytokines such as IL-4 and IL-6, which promote maturation and clonal proliferation of B cells. Treg cells can suppress the immune response in the host, including inhibiting the activation of NK cells and the cytotoxic function of CD8 + T cells (76). Kono et al. (77) confirmed that as a kind of Treg, the high expression of CD4 (+) CD25 high T cells was closely related to the worse prognosis of gastric and esophageal cancer. Interestingly, the level of CD4 (+) CD25 high T cells decreased significantly after patients with GC undergoing radical resection. Zhuang et al. (78) revealed that the overexpression of IL-22(+) CD4(+) T cells and Th22 cells were associated with tumor progression and predicted reduced overall survival. In addition, CD8 (+) T cells that produce IL-7 can promote the progression of GC cells by promoting chemotaxis of myeloid-derived suppressor cells (79).

### Features of Gastric TME

*Helicobacter pylori* (HP) was confirmed to be associated with approximately 75% of GC events worldwide as a group 1 carcinogen (80, 81). HP infection is able to significantly affect and change the microenvironment of the stomach by inducing chronic inflammation of the stomach, while the inflammatory response will promote EMT of GC through a variety of mechanisms. Alternatively, HP cytotoxin-associated gene A (CagA) can change many signaling pathways of the host cell by inducing DNA damage and changing DNA methylation, and then induce the production of GC EMT. In addition, it also induces the generation of GC EMT by down-regulating the expression of E-cadherin and up-regulating the expression of vimentin and twist (82–84). CagA is also able to activated NFκB and STAT3 signals and increased the expression of SNAIL1 protein, which is closely related to CAFs activation and EMT in GC cells (85). Zhang et al. (86) found that HP infected gastric epithelial cells could activate and recruit MSCs and promote the conversion of MSCs into CAFs, which may promote the EMT of gastric epithelial cells. Krzysiek-Maczka et al. (87) found that HP strains are not only able to induce EMT of normal rat gastric epithelium cells, but also induce differentiation of rat normal gastric fibroblasts into CAFs.

An acidic microenvironment is a basic characteristic of the metabolic environment of tumor tissue. Tumor cells utilize glycolysis to create an acidic microenvironment conducive to invasion and metastasis (88–90). Current studies suggest that both glycolysis and oxidative phosphorylation may be the metabolic pathways of CSCs (91, 92), but it is undeniable that the acidic microenvironment favors the progression of malignant tumors and the emergence of CSCs (93). For most normal cells, living in an acidic microenvironment is harmful, while tumor cells can survive in the acidic environment and rely on the acidic microenvironment to maintain their ability to grow and proliferate rapidly (94). An acidic microenvironment and acid-sensitive ion channel 1a (ASIC1a) were confirmed to promote the proliferation and migration of GC cells. Chen et al. (95) found that the expression of ASIC1a was significantly increased in GC tissues with postoperative metastasis compared with GC tissues without postoperative metastasis and non-tumor tissues. In addition, down-regulation of ASIC1a expression by silencing ASIC1a *via* shRNA was able to reduce the migration and invasion of GC cells. However, the regulatory mechanism of the acid-base microenvironment on tumor cell growth and metastasis is still unclear, and regulating the pH value of the GC microenvironment may be an effective measurement to kill GC cells.

## Interaction Between CSCs and TME

**Figure 3:**
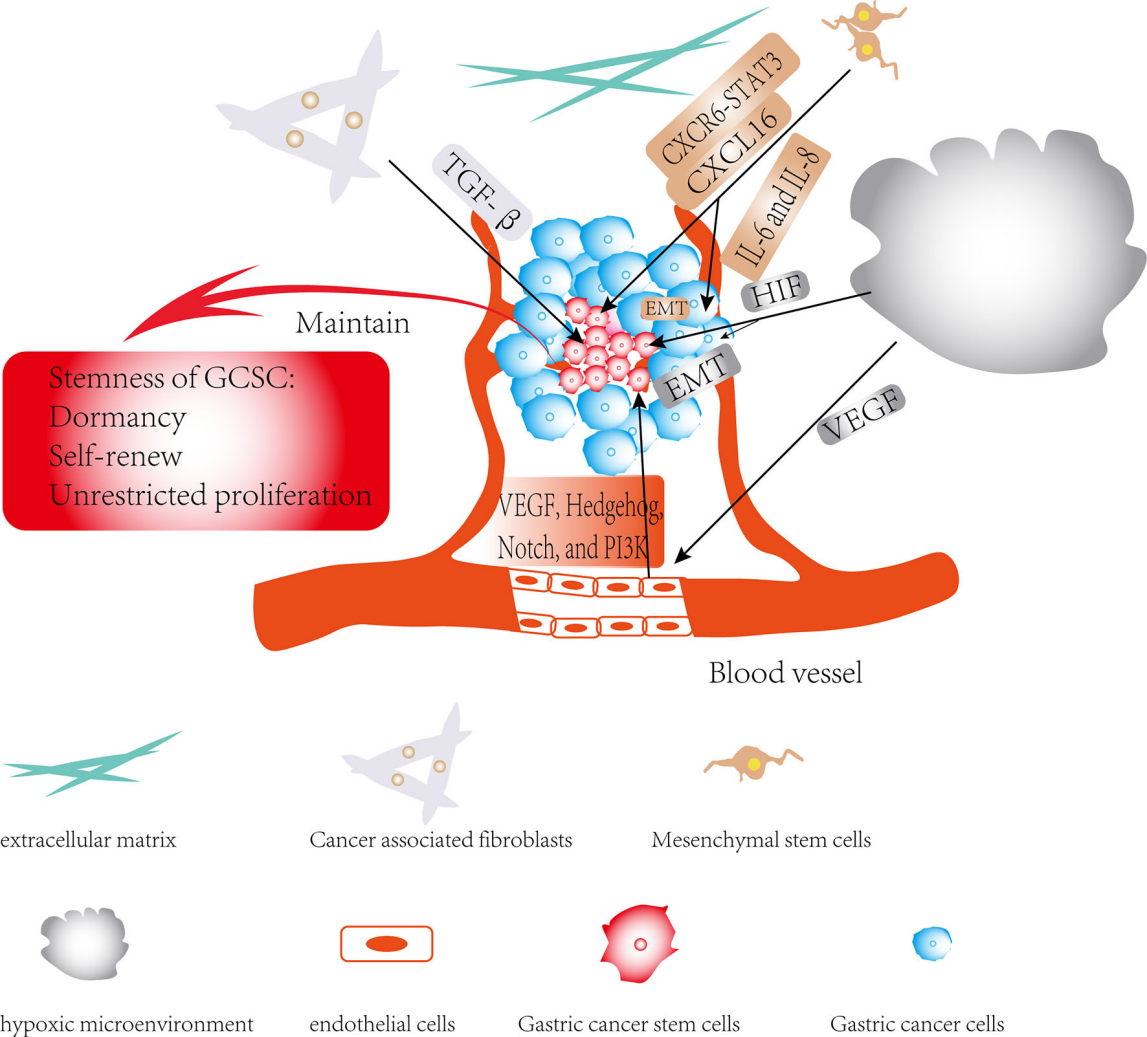
Regulatory role of TME in gastric cancer stem cells (GCSCs): The effects of members of the TME on GCSCs are shown by black arrows; the role of TME in maintaining the stemness of GCSCs is shown by red arrows.

With the continuous deepening of research in cancer, it has been slowly discovered that there is a complex dialogue between CSCs and the TME during tumor development. TME can not only affect the self-renewal ability of CSCs, but it may also induce the transformation of its surrounding non-tumor stem cells into CSCs (96). The TME surrounding CSCs can secrete cytokines such as hypoxia-inducible factor (HIF) and IL-1β, activate related signaling pathways, and participate in CSCs invasion and metastasis by inducing angiogenesis, induction of EMT and protecting CSCs from being attacked by the host’s immune system. CSCs may also rely on their surrounding microenvironment to maintain their stem cell characteristics, such as self-renewal, dormancy and multi-lineage differentiation potential (**Figure 3**) (6, 97–101). Furthermore, the CSCs can also affect and modify the nature of the microenvironment. There is increasing evidence that CSCs are able to recruit and activate special types of cells such as MSCs to form a microenvironment suitable for the survival of CSCs, which is commonly known as the CSCs niche. The CSCs niche consists of stromal cells, immune cells, extracellular matrix (ECM), a vascular network and soluble factors (102). At present, there is little literature on exploring the interaction between CSCs and the TME in GC, and this paper only summarizes the existing literatures.

Recent studies have shown that CSCs reside in a vascular microenvironment that provides a suitable environment for the long-term growth of CSCs and maintenance of their stem-like properties. And vascular endothelial cells also play a crucial role in maintaining the stemness and self-renewal of CSCs. Some studies have shown that anti-vascular therapy (such as targeting VEGF) is effective in reducing the counts of CSCs and inhibiting tumor growth (11, 103). These studies demonstrate the critical role of perivascular microenvironment and vascular endothelial cells for the maintenance of the stemness of CSCs. Bevacizumab is a humanized monoclonal antibody that effectively prevents VEGF from binding to VEGFR-1, VEGFR-2, thereby inhibiting vascular endothelial cell proliferation and angiogenesis (104). Several studies have also demonstrated that targeted inhibition of the vascular microenvironment in GC can effectively inhibit the progression of GC. To explore the efficacy of bevacizumab in GC, a study *in vitro* suggested that the use of bevacizumab before chemotherapy can effectively improve the tumor control rate and reduce the tumor volume (105). In addition, it has been found that the combination of bevacizumab, docetaxel/oxaliplatin/5-FU can increase the R0 resection rate of initially unresectable GC, indicating that bevacizumab is effective in the treatment of advanced gastric cancer (106). In addition, treatment with trastuzumab in combination with VEGF-Trap can effectively inhibit the development of HER2-overexpressing GC (107).

Although the perivascular microenvironment of CSCs is increasingly studied, recent studies have found that many CSCs also reside inside tumors that are far from blood vessels and in a hypoxic state, which is known as the hypoxic microenvironment of CSCs. It has been reported that hypoxia can maintain the stemness of CSCs and induce tumor cells to become biologically more aggressive such as invasion, metastasis and therapeutic resistance. These effects are mainly attributed to HIF (108, 109). However, the hypoxic microenvironment and perivascular microenvironment of CSCs are not hostile, and the hypoxic microenvironment is also able to promote angiogenesis. In addition, both endothelial cells and CSCs can produce VEGF to stimulate angiogenesis under a hypoxic environment. Hypoxia can protect CSCs from DNA-damaging agents including chemotherapy and radiation therapy, and also promote the survival and EMT of CSCs through reactive oxygen species (ROS)-activated stress pathway and TGF-β signaling pathway. And hypoxia possess the ability of maintaining the self-renewal, tumorigenicity and the undifferentiated state of CSCs (110, 111). GCSCs microenvironment can also affect the characteristics of stem cells by regulating the expression of some transcription factors and tumor-related genes, and then affect the biological characteristics of tumor cells. For instance, Hasegawa et al. (42) confirmed that TGF-β could blocks the effects of CAFs on GCSCs, reducing the number of SP cells in GCSCs and the level of surface marker expression of GCSCs. Maeda et al. (112) found that Wnt5a gene involved in stem cell niche may promote the invasive properties of GCSCs. It was reported that hypoxia is able to confer a stem cell-like phenotype on GC, enhancing drug resistance, radiation resistance, and EMT of GC cells (113–115). However, the specific mechanism of hypoxia affecting the characteristics of CSCs is still unclear and needs further research.

## Anti-Cancer Therapy Targeting GC Microenvironment and GCSCs

### Strategies for Targeting GC Microenvironment

Most of the existing clinical drugs for GC treatment are aimed at the characteristics of rapid proliferation of tumor cells, such as cytotoxic chemotherapeutic drugs. Recently, with the in-depth understanding of the role and mechanism of TME in tumor progression, great progress has also been made in the study of targeted TME therapy for tumors including GC. Here, we will show some strategies based on targeting the GC microenvironment for the treatment of GC.

Immune checkpoints are pathways that inhibit the immune system through interactions between ligand and receptor and can regulate the immune system to avoid damage to normal cells and tissues. Cancer cells, on the other hand, evade the surveillance and killing of the immune system by using immune checkpoints, leading to the continuous progression of cancer. Recently, anti-programmed cell death 1 (anti-PD-1) and anti-PD-1 ligand (anti-PD-L1) antibodies with good anti-tumor effect have gradually attracted much attention (116). Briefly, PD-1 is a transmembrane protein on the surface of T cells, and T cells will not kill cancer cells when PD-1 binds to PD-L1 expressed on the surface of cancer cells. Anti-PD-1 antibody can bind to PD-1 expressed on T cells, while anti-PD-L1 antibody can bind to PD-L1 expressed on cancer cells, thus preventing PD-1 expressed on T cells from binding to PD-L1 expressed on tumor cells, allowing T cells to kill cancer cells (117). For instance, pembrolizumab (anti-PD-1 antibody) has been shown a significant survival benefit in the treatment of GC in many studies and has been approved by the Food and Drug Administration (FDA) as a third-line treatment for PD-L1-positive GC patients as well as patients with unresectable or metastatic, highly microsatellite instability or mismatch repair-deficient (118).

CAFs have already been recognized to play a key role in promoting GC progression. Therefore, drugs that inhibit the function of CAFs may potentially prevent the progression of GC. Wang et al. (119) cultured gastric CAFs (GCAFs) and gastric normal fibroblast (GNF) with BGC-823 human GC cells, respectively. The results of this study showed that GCAFs could significantly promote the proliferation, migration and invasion of BGC-823 cells by down-regulating microRNA-214 and up-regulating microRNA-301a compared with GNFs. And astragaloside IV, a main component of nontoxic Chinese herb, can inhibit the proliferation and invasion of GC by inhibiting the pathological function of CAFs through regulation of microRNA expression. Ferroptosis is a form of regulatory cell necrosis induced by lipid peroxidation (lipid-ROS), iron and reactive oxygen species. Zhang et al. (120) demonstrated that exosomal microRNA-522 secreted by CAFs was able to inhibit ferroptosis in GC cells by inhibiting arachidonate lipoxygenase 15 and reducing lipid-ROS production. In addition, cisplatin and paclitaxel were also demonstrated to be able to increase the secretion of microRNA-522 from CAFs by activating the USP7/hnRNPA1axis, resulting in decreased chemosensitivity of GC cells. And in another study, Uchihara et al. (121) showed Annexin A6 in extracellular vesicles (EV) from CAFs induced drug resistance of GC by activation of β1 integrin-focal adhesion kinase (FAK)-YAP. They also revealed that inhibition of FAK or YAP was able to effectively attenuate drug resistance of GC in a mouse model of peritoneal metastasis. Shen et al. (122) showed HP infection can increase expression of vascular adhesion molecule 1 (VCAM1) in CAFs of GC by activating the JAK/STAT1 signaling pathway, and the expression level of VCAM1 is positively correlated with the progression of GC and the poor prognosis of patients with GC. In addition, the interaction between CAFs-derived VCAM1 and integrin αvβ1/5 can promote the invasiveness of GC *in vivo* and *in vitro*. These findings facilitate us to further understand the mechanism of how CAFs promote GC progression and drug resistance, and provide potential targeted therapeutic strategies for GC treatment and overcoming GC drug resistance.

As an essential component of TME, TAMs are considered to be closely associated with the progression, metastasis and drug resistance of GC. Among TAMs, those of the M2 type are responsible for inhibiting T cell function and promoting tumor growth. Therefore, several potential therapeutic strategies have been proposed to work on eradicating M2 TAMs or converting M2 TAMs into M1 TAMs. Miao et al. (123) performed an immunohistochemical analysis of STING expression in 200 pairs of GC cells and its surrounding normal tissues and detected the effects of STING on cancer cell apoptosis and T cell differentiation by flowcytometry. They also verified the results in a spontaneous GC model of p53+/- mice and cell line-based xenografts. The results of this study suggest that down-regulation of STING expression is able to promote TAMs polarizing into the M1 as well as induce apoptosis in GC cells through the IL6R-JAK-IL24 pathway. Zheng et al. (124) found that M2-type TAMs were able to promote cisplatin resistance in CG cells. Further analysis using the microRNA profiles assay confirmed that exosomal microRNA-21 derived from M2-type TAMs conferred cisplatin resistance in GC cells. Wang et al. (125) confirmed *in vitro* that the expression levels of Legumain in TAMs were positively correlated with the proliferation and angiogenesis of GC. Further experiments *in vivo* also confirmed that GC cells injected with Legumain-knockdown TAMs showed slower growth and less angiogenesis compared with GC cells injected with TAMs. These studies showed that targeting exosomes associated with TAMs may be a promising new therapeutic strategy for the therapy of GC and overcoming drug resistance of GC.

Another important component in TME, MSCs plays a key role in the progression of GC and may be a promising therapeutic target for GC. Accumulating evidence indicates that MSCs contribute to progression and chemotherapy resistance of GC by secreting soluble molecules and regulating various signaling pathways. GC-MSCs have been confirmed to promote immune escape by secreting IL-8 and can induce the expression of PD-L1 in GC cells. Sun et al. (126) further confirmed that GC-MSCs were able to enhance the stemness and self-renewal of GC cells through PD-L1, leading to chemoresistance of GC. It has also been confirmed that MSCs can promote the stemness and chemoresistance of GC cells both *in vivo* and *in vitro* through fatty acid oxidation (FAO). And FAO inhibitors have been demonstrated to reduce MSCs induced resistance to FOLFOX chemotherapy regimens in GC cells. These results of this study suggested that FAO was a key factor in MSC-induced stemness and chemoresistance of GC cell and inhibitors targeting FAO combined with conventional chemotherapy regimens may be a promising therapeutic strategy to overcome GC chemoresistance (127). In addition, exosomes secreted by MSC are responsible for 5-FU resistance in GC cells both *in vivo* and *in vitro*. Ji et al. (128) revealed that MSC-derived exosomes can prevent 5-FU -induced apoptosis of GC cells and increase the expression of multi-drug resistance (MDR)-associated proteins, such as MDR by activating the calcium/calmodulin-dependent protein kinases (CaM-Ks) and Raf/MEK/ERK signaling pathway. And inhibition of CaM-Ks/Raf/MEK/ERK signaling pathway is also able to inhibit GC chemoresistance induced by MSC-exosomes. These findings suggest that targeting MSCs-related soluble molecules and signaling pathways combined with conventional chemotherapy may provide a promising new strategy to overcome the resistance of GC cells to conventional chemotherapy.

At present, surgical resection and chemoradiotherapy are the main therapeutic strategies for the treatment of GC, and molecular targeted drugs are an emerging therapeutic strategy for the treatment of GC. Increasing studies have shown that targeting key signaling pathways and molecules of the TME in GC may be a new promising therapeutic strategy for the treatment of GC. While knowledge is emerging regarding preclinical studies of the GC microenvironment, data from clinical studies on targeting the GC microenvironment in the therapy of GC is still limited. Thus, there is still a long way to go before the therapy of targeting the GC microenvironment can be applied in the treatment of GC. The underlying specific mechanisms involved in TME affecting the progression, recurrence, metastasis and drug resistance of GC remain poorly understood and further studies are still warranted.

### Strategies for Targeting GCSCs

GCSCs are considered to be responsible for the development, recurrence, metastasis and drug resistance of GC. Therefore, therapeutic strategies for the targeted elimination of CSCs are considered to be one of the promising approaches for the treatment of GC. However, there are methodological dilemmas in the current therapeutic strategy for targeting CSCs, including the lack of detection methods to specifically identify and isolate CSCs from tumor cells. This paper summarizes the current promising strategies for targeting GCSCs.

There are some specific surface markers in the surface of GCSCs. Targeting these specific surface markers is an important way to kill GCSCs and improve the prognosis of GC. Many investigators have searched for specific surface markers of GCSCs for a long time, and CD44 has been confirmed in some studies as a specific surface marker of GCSCs. Yao et al. (129) developed a gastric CSCs-specifically targeting drug delivery system (SAL-SWNT-CHI-HA complexes) that can inhibit the self-renewal ability of CD44 (+) cells in serum-free medium, which in turn reduces the formation of GCSCs. Through the eradication of GCSCs, it can effectively eradicate GC cells and block the migration and invasion of GC cells. Liang et al. (130) developed a nanoprobe against CD44v6 (a surface marker of GCSCs), which specifically targets GCSCs. In orthotopic and subcutaneous xenograft models of GC, this nanoprobe actively targets the vascular system of GC and significantly inhibits the growth of GC at 4 hours post-injection. In addition, aberrant activation of signaling pathways is present in GCSCs, and a therapeutic strategy for targeting key signaling pathways in GCSCs appears theoretically feasible in reducing GCSCs and improving GC patient prognosis. GSI, a γ-secretase inhibitor IX, was reported to be capable of inhibiting the proliferation, migration, invasion, and tumor sphere formation of CD44 (+) GCSCs by inhibiting the Notch signaling pathway (131). Feng et al. (132) found that in a GCSCs model, pantoprazole was able to increase the therapeutic sensitivity of GC to 5-fluorouracil, decrease the capacity of generating tumor spheres and the expression levels of GCSCs markers such as CD44, CD24, and Lgr5 by inhibiting the EMT/β−catenin pathways. In a study (133), CD44 (+) GC cells were shown to be resistant to 5-FU and cisplatin chemotherapy. In this study, Hedgehog (HH) signaling played an important role in maintaining the stem-like properties of CD44 (+) GC cells, and inhibition of HH could increase the sensitivity of CD44 (+) GC cells to chemotherapy. Mao et al. (134) found that activation of Wnt signaling can promote the self-renewal and proliferation of GCSCs, and salinomycin can inhibit the growth of GC by inhibiting Wnt signaling in GCSCs. The chimeric 5/35 adenovirus-mediated Dickkopf-1(Ad5/35-DKK1) that could effectively attenuate Wnt signaling of GCSCs was developed by Wang et al. (135). And in preclinical experiments, Ad5/35-DKK1 was demonstrated to be able to inhibit the invasion of CD44 (+) GC cells. Although research on targeting GCSCs is accumulating, more studies, especially clinically relevant studies, are needed to demonstrate the clinical significance of therapeutic strategy for targeting CSCs.

## Conclusion and Future Perspectives

The TME is a complex biological system composed of a variety of cells, extracellular matrix and biological molecules. It is closely related to tumorigenesis, invasion, metastasis, and immune evasion of GC cells by secreting a variety of factors and regulating signaling pathways. CSCs are a small population of tumor cells with stem-like characteristics, which also play a crucial role in the progression, metastasis and drug resistance of GC. On the one hand, TME and CSCs may synergistically contribute the progression of GC. On the other hand, TME and CSCs may mutually promote each other. And future studies to investigate the relationship among them will provide a new idea for the cancer progression and novel therapeutic targets in GC. Because various current targeted therapeutic strategies for GC are mainly to kill non-CSCs, and the residual CSCs of GC after current therapy are implicated in tumor recurrence and metastasis. Consequently, the development of therapy targeting CSCs is warranted for the effective treatment of GC. In addition, studies focusing on targeting TME in the progression and drug resistance of GC have also confirmed that TME may provide new targets for the treatment of GC. In the future, further study on the microenvironment of CSCs in GC should be carried out to clarify the different components and functions of the microenvironment of CSCs in GC. Which will be helpful to understand the mechanism underlying the pathogenesis of GC, develop new therapy to kill CSCs in GC and change GC microenvironment, leading to promote the clinical treatment of GC.

## Author Contributions

Literature review, data analysis, and manuscript preparation were performed by YY. W-JM and Z-QW contributed for the study conception, design, and revision. All authors contributed to the article and approved the submitted version.

## Conflict of Interest

The authors declare that the research was conducted in the absence of any commercial or financial relationships that could be construed as a potential conflict of interest.

## Publisher’s Note

All claims expressed in this article are solely those of the authors and do not necessarily represent those of their affiliated organizations, or those of the publisher, the editors and the reviewers. Any product that may be evaluated in this article, or claim that may be made by its manufacturer, is not guaranteed or endorsed by the publisher.

## Abbreviations

Ad5/35-DKK1, Adenovirus-mediated Dickkopf-1; ALDH, Aldehyde dehydrogenase; ALOX15, Arachidonate lipoxygenase 15; anti-PD-1, Anti-programmed cell death 1; anti-PD-L1, Anti-PD-1 ligand; ASIC1a, Acid-sensitive ion channel 1a; BM-MSCs, Bone marrow-derived MSCs; CAFs, Cancer associated fibroblasts; CagA, Cytotoxin-associated gene A; CaM-Ks, Calcium/calmodulin-dependent protein kinases; CSCs, Cancer stem cells; CTL, Cytotoxic T cells; DGCs, Diffuse-type gastric cancers; ECM, Extracellular matrix; EMT, Epithelial-mesenchymal transformation; EpCAM, Epithelial cellular adhesion molecule; FAK, Focal adhesion kinase; FAO, Fatty acid oxidation; FAP, Fibroblast activating protein; FDA, Food and Drug Administration; FGF, Fibroblast growth factor; GC, Gastric cancer; GCAFs, Gastric CAFs; GC-MSCs, GC-derived MSCs; GCSCs, Gastric CSCs; GEO, Gene Expression Omnibus; GNF, Gastric normal fibroblast; HH, Hedgehog; HIF, Hypoxia-inducible factor; HP, Helicobacter pylori; IL, Interleukin; MCP-1, Monocyte chemoattractant protein-1; MDR, Multi-drug resistance; MSCs, Mesenchymal stem cells; NK, Natural killer; PD-1, Programmed cell death 1; PDGF, Platelet-derived growth factor; PD-L1, PD-1 ligand; RHBDF2, Rhomboid 5 Homolog 2; ROS, Reactive oxygen species; SCID, Severe combined immunodeficiency; SP, Side population; TAMs, Tumor-associated macrophages; TCGA, The Cancer Genome Atlas; TGF, Transforming growth factor; Th, Helper T cells; TILs, Tumor-infiltrating lymphocytes; TME, The tumor microenvironment; TNF, Tumor necrosis factor; Treg, Regulatory T cells; VCAM1, Vascular adhesion molecule 1; VEGF, Vascular endothelial growth factor.
